# Cytoprotective Effect of Growth Factors Derived From Platelets on Corticosteroid-Treated Primary Anterior Cruciate Ligament-Derived Stromal Cells and Chondrocytes

**DOI:** 10.7759/cureus.65566

**Published:** 2024-07-28

**Authors:** Vijay Sharma, Ulka Sakhalkar, Pratiksha Nadkarni, Rashmi Mishal, Dinesh Parandhaman, Kirti Vichare, Anjalina Francis, Mudit Khanna, Mohit Kukreja, Anuka Sharma

**Affiliations:** 1 Department of Regenerative Medicine, Wockhardt Regenerative Pvt. Ltd., Mumbai, IND; 2 Orthopaedic Surgery, Wockhardt Hospital, Mumbai, IND

**Keywords:** acl-derived stromal cells, musculoskeletal disease, platelet growth factors, glucocorticoids, immunocytochemistry, chondrocytes

## Abstract

Background

The use of corticosteroids, such as methylprednisolone, for pain management is a common clinical practice. However, it is well known that corticosteroids induce toxicity in anterior cruciate ligament (ACL)-derived stromal cells and chondrocytes. Growth factors from platelets have anti-inflammatory effects that can potentially limit the cytotoxic effects of corticosteroids. In this study, we explored the role of growth factors obtained from the Ossinext^TM^ kit (commercially available Wockhardt growth factor concentrate (GFC) kit) in recovering methylprednisolone-induced cell damage.

Methodology

Primary ACL-derived stromal cells and chondrocytes were isolated from human ligament tissue and articular cartilage, respectively, and characterized by immunophenotyping, gene expression analysis, and immunostaining. GFC obtained from Ossinext^TM ^kit was used for the experiments.

The ACL-derived stromal cells and chondrocytes were treated with methylprednisolone, alone or in combination with GFC. Cell viability was measured by the neutral red uptake assay. Changes in cell morphology and collagen pattern were observed microscopically by H&E staining and immunostaining, respectively. Cell proliferation was assessed by cell migration assay, and the cell ultra-structure was analyzed using transmission electron microscopy.

Results

Methylprednisolone was found to induce cytotoxicity, altered cell morphology, reduced cell proliferation, and organelle damage in both ACL-derived stromal cells and chondrocytes. GFC obtained from the Ossinext^TM^ kit was able to restore cell viability and reverse the cell structure damages induced by methylprednisolone. GFC was found to recover and protect the cells, both when used in combination with steroids and when used after the steroid treatment.

Conclusions

The results indicate that GFC may be clinically beneficial when used in combination with steroids to mitigate their adverse effects.

## Introduction

Musculoskeletal disorders that affect the joints, ligaments, and tendons result in diseases such as osteoarthritis and osteoporosis, which lead to health ailments like long-term pain, discomfort, and limited mobility, as well as socioeconomic problems [1,2]. Musculoskeletal pain that arises from these diseases is managed by corticosteroid injections along with local anesthetics, which provides short-term relief along with clinical adverse events and cell damage [2,3]. Clinically, post-injection flare, steroid-induced arthropathy, tendon rupture, intra-articular infection, and hypersensitivity reaction are some of the reported adverse events due to corticosteroids [2]. Connective tissue being hypocellular and hypovascular in nature has poor regenerative abilities. Therefore, the use of corticosteroids may inhibit their reparative mechanism [4]. Corticosteroid-induced cell damages were studied in vitro using cells derived from the anterior cruciate ligament (ACL) (ligaments), tenocytes (tendons, ligaments), and chondrocytes (cartilage) after treatment with corticosteroids, like methylprednisolone, or dexamethasone. Dexamethasone-treated ACL cells have been shown to increase apoptosis, increase in tendency to calcify, induce ACL degeneration by endoplasmic reticulum stress, reduce cell proliferation, and inhibit collagen accumulation [5,6]. Similarly, glucocorticoid treatment causes chondrotoxicity evinced by reduced cell viability and decreased CD44 expression in chondrocytes [7]. Orthobiologics are substances derived from natural and biological sources that are often used in orthopedic medicine to promote tissue repair, reduce inflammation, and enhance the healing process in conditions related to bones, joints, and soft tissues. Growth factors, stem cells, bone grafts, and other biologically active substances that stimulate healing and regeneration are examples of orthobiologics. Several studies have demonstrated the efficacy of these orthobiologics and could possibly be used as an alternative to corticosteroids [8]. Platelet-rich plasma (PRP), an autologous minimally invasive, growth factor enriched therapy, is commonly used for tissue regeneration in diseases like plantar fasciitis, lateral epicondylitis, and knee osteoarthritis, for tendon and cartilage repair [9].

PRP was shown to exert cytoprotective effects by increasing cell proliferation, migration, and suppression of apoptosis in both hypoxic-induced and normal ACL cells [10,11]. In human chondrocytes and tenocytes, the addition of PRP to corticosteroids or local anesthetics has been shown to increase cell proliferation [12]. A recent systematic review underscored the role of PRP using studies from in vitro models to increase cell proliferation, migration, viability, and total collagen production in both ligament- and tendon-derived cells [13].

Growth factor concentrate (GFC) is a modified PRP containing acellular preparation enriched with growth factors that is prepared by platelet activation. GFC preparation ensures maximal release of growth factors from platelet granules while eliminating red and white blood cells in the final output [14]. GFC therapy has been used in orthobiologics with significant regenerative effects [14,15]. The above studies, including our experience in using PRP-derived products like GFC, underline the cytoprotective effects of PRP in musculoskeletal injuries.

In this study, we demonstrate the cytoprotective effects of GFC in supporting the reparative activities of musculoskeletal disorders by mitigating cell toxicity caused by methylprednisolone in ACL-derived stromal cells and cartilage-derived chondrocytes.

## Materials and methods

This study was approved by the Wockhardt Hospital Institutional Ethics Committee (approval no. WH-IRB/STUDY/2019-07), and informed consent was received from all the participants.

GFC preparation

GFC from 10 healthy individuals was prepared using Ossinext^TM^ GFC kit (Wockhardt Regenerative Pvt. Ltd, Mumbai, India) after obtaining informed consent. The sampled blood was processed to obtain GFC as described earlier [14].

The resulting GFC was pooled and analyzed to quantify the levels of growth factors, such as transforming growth factor-beta (TGF-β), platelet-derived growth factor (PDGF), vascular endothelial growth factor (VEGF), interleukin-1 receptor antagonist (IL-1RA), and epidermal growth factor (EGF), using the enzyme-linked immunosorbent assay (ELISA) technique. The ELISA assays were performed by following the manufacturer’s protocol of standard kits available from R&D Systems (Cat. no. DB100C, DBB00, DVE00, DRA00B, DEG00).

Isolation of ACL-derived stromal cells and chondrocytes

The tissue samples for this study were collected from patients who underwent ACL reconstruction surgery and total knee arthroplasty. Fragments of ACL were obtained from six donors (mean age 65 ± 10 years) undergoing ACL reconstruction surgery. Human articular cartilage samples were obtained from four donors undergoing total knee arthroplasty (mean age 70 ±10 years). In both sample types, the patients chosen had no other comorbidities and were not subjected to any corticosteroid treatment six months prior to the surgery.

ACL-derived stromal cells were isolated from the ACL fragments, which were washed with phosphate-buffered saline (1X PBS) (CellClone), minced, and subjected to enzymatic digestion with 3% type I collagenase (Gibco) for two hours at 37°C [16,17]. The released cells were strained through a 70μm cell strainer (Falcon) and centrifuged. The obtained cell isolate was expanded in a complete culture medium consisting of Dulbecco's Modified Eagle's Medium (DMEM)-High Glucose (HG) (MP Biomedicals) and DMEM-F12 (Gibco) in a 1:1 proportion along with 20% fetal bovine serum (Gibco), and 1% penicillin/streptomycin (MP Biomedicals). Under standard culture conditions (37°C humidified chamber with 5% CO_2_), adherent cells were expanded to sub-confluence up to passage 3 for further analysis.

Chondrocytes were isolated using articular cartilage samples, which were washed vigorously in 1X PBS to remove impurities. These samples were minced into small pieces (1-3 mm) and subjected to enzymatic digestion with 0.2% type I collagenase (Gibco) for 16 hours at 37°C on a rocker incubator [18]. The released chondrocytes were passed sequentially through 100 μm and 70 μm cell strainers (Falcon) and centrifuged. The isolated cells were expanded under standard culture conditions (37°C humidified chamber with 5% CO_2_) using DMEM-F12 (Gibco) along with 20% fetal bovine serum (Gibco), and 1% penicillin/streptomycin (MP Biomedicals) up to passage 3 for experimental analysis.

Phenotyping of ACL-derived stromal cells and chondrocytes using flow cytometry

Phenotypes of isolated ACL-derived stromal cells and chondrocytes were assessed at P4 as reported earlier [16-20]. Stromal cells isolated from ACL were identified by staining of 1 × 10^6^ cells with anti-human CD44 (FITC), CD105 (PERCP), CD90 (PE), CD45 (FITC), CD31 (FITC) (BD Biosciences), and chondrocytes were phentoyped using 1.4 × 10^6^ cells stained with anti-human CD44 (FITC), CD45 (FITC), CD49c (PE), CD73 (APC), CD90 (PE), CD105 (APC), CD151 (PE), HLA-ABC (PE-CY5), and HLA-DR (FITC) (BD Biosciences), encompassing positive and negative markers. Cells were incubated with the antibodies at 4°C for one hour, washed with 1X PBS, and examined using a BD FACSCanto™ II system flow cytometer. The viability of cells was determined using 7-aminoactinomycin D (7-AAD). About 30,000 events were captured for each sample, and the percentage of positive cells was calculated using the FlowJo software version 10.6 (Tree Star, BD Life Sciences, United States).

Immunocytochemistry

The phenotype of ACL-derived stromal cells and chondrocyte was assessed at P4 using polyclonal antibodies collagen type I (Invitrogen; Cat. no. PA1-26204), scleraxis A (Invitrogen; Cat. no. PA5-115874), and tenascin C (Merck; Cat. no. HPA004823) as per manufacturer instructions. These markers were shown to be expressed by both ACL-derived cells and chondrocytes [20]. A total of 18,000 cells per well were plated in 48-well culture plates and fixed in 10% buffered formalin at 50-60% confluency. The staining was performed as reported earlier with modifications [21].

Briefly, fixed cells were peroxidase quenched and blocked using buffers from Peroxidase IHC Detection Kit (Thermo Fisher Scientific Inc., United States; Cat. no. 36000). Primary antibodies were used at 1:50 dilution, followed by the addition of an anti-rabbit secondary antibody with HRP (Merck, United States) for 60 minutes at room temperature. The signal was detected using chromogenic substrate 3, 3′-diaminobenzidine (DAB) and captured using an Olympus inverted microscope (Olympus Corp., Japan) equipped with Magnus MagCam DC-5 CMOS camera (‎Magnus, United States).

Reverse transcriptase polymerase chain reaction

Total RNA was extracted from cultured ACL-derived stromal cells and chondrocytes at P4 using GeneJET RNA Purification Kit (Thermo Fisher Scientific Inc.; Cat. no. K0371) as per the manufacturer's instructions. Single-stranded cDNA was synthesized from 0.5 µg of RNA using random hexamers from RevertAid First Strand cDNA Synthesis Kit (Thermo Fisher Scientific Inc.; Cat. no. K1622). The isolated RNA was quantified using a Thermo Fisher Scientific™ μDrop™ plate. Genes specific for ACL-derived stromal cells (Genex Life Sciences, India) and chondrocyte (Eurofins Genomics LLC, United States) lineages were assessed using the primers given in Table 1. These markers were used in earlier studies for identifying ACL-derived stromal cells and chondrocytes [16-21].

**Table 1:** Reverse transcription–polymerase chain reaction primer sequences for ACL-derived stromal cells and chondrocytes ACL: anterior cruciate ligament

Human genes	Forward primer sequences (5’ to 3’)	Reverse primer sequences (5’ to 3’)	Amplicon size (bp)
Housekeeping gene			
Glyceraldehyde-3-phosphate dehydrogenase (GAPDH)	CCATGAGAAGTATGACAACAGCC	CCTTCCACGATACCAAAGTTG	496
ACL-derived stromal cell lineage			
Collagen type I alpha 1 chain (COL1A1)	TGACCTCAAGATGTGCCACT	ACCAGACATGCCTCTTGTCC	197
Tenascin C (TNC)	TTCACTGGAGCTGACTGTGG	TAGGGCAGCTCATGTCACTG	223
Scleraxis bHLH transcription factor (SCX)	GCTACATCTCGCACCTGGG	TGCAGATCTGTTTGGGCTGG	144
Chondrocyte lineage			
Collagen type I alpha 1 chain (COL1A1)	AGAGTGGAGAGTACTGGATTGA	GTTGGGATGGAGGGAGTTTAC	604
Collagen type II alpha 1 chain (COL2A1)	GTCCTCTGCGACGACATAATC	CATCAAATCCTCCAGCCATCT	382
Aggrecan core protein (ACP)	CAGAATGGGAACCAGCCTATAC	TCAAGGTGTCCTGAAACATCTC	424
Human transcription factor SOX-9 (SOX-9)	ACCTATCCAAGCGCATTACC	AAGGCAGCTCCTCCTTAAATC	536
Collagen type X alpha 1 chain (COL10A1)	GACCCAAGGACTGGAATCTTTA	CTGAGAAAGAGGAGTGGACATAC	274

Reagent control containing a reaction mixture with nuclease-free water instead of a cDNA template prepared in parallel was used as the PCR-negative control for all the lineage genes. The PCR products were separated on 2% agarose gel stained with Nex-Gen Green Stain (Puregene) and visualized using an LED book transilluminator.

Multipotent differentiation of ACL-derived stromal cells in vitro

The multipotency of primary ACL-derived stromal cells was assessed by inducing the cells toward adipocyte, chondrocyte, and osteocyte lineages. For adipogenic and osteogenic induction, cells at P3 were seeded in a 24-well plate in a complete culture medium and allowed to grow to confluency. The confluent monolayers were then treated with StemPro™ Adipogenesis (Gibco; Cat. no. A1007001) and StemPro™ Osteogenesis (Gibco; Cat. no. A1007201) induction media, respectively. Cells grown with a complete culture medium served as controls. The medium was changed according to the manufacturer’s instructions in both groups for up to 21 days.

For chondrogenic induction, cells at P3 were seeded as micro mass cultures in six-well plates, followed by a careful addition of the StemPro™ Chondrogenesis induction medium (Gibco; Cat. no. A1007101) in the test well and regular culture medium in the control well, without disturbing the micromasses. The medium was changed according to the manufacturer’s instructions in both groups until the cells showed signs of induction [16,17].

Oil-Red-O, Alizarin red, and Safranin-O staining were used to confirm adipogenic, osteogenic, and chondrogenic inductions, respectively [16,17]. Imaging was performed using an Olympus CKX53SF inverted microscope, equipped with a Magcam DC 5 camera and MagVision 4.6 software.

Neutral red uptake (NRU) cytotoxicity assay

The effect of methylprednisolone and GFC on the viability of ACL-derived stromal cells and chondrocytes was studied using the NRU assay as reported earlier [22]. Briefly, ACL-derived stromal cells and chondrocytes were seeded in 96-well plates at a density of 5000 cells per well and treated with methylprednisolone and GFC in various combinations. Untreated cells were used as control, while DMSO at various concentrations (1%, 3%, 7%, and 10%) was used as positive control. The assay was performed in triplicates in four biological replicates of each cell type. After treatment for 24 hours with combinations of methylprednisolone and GFC, neutral red dye (50 µg/ml) was added and cells were incubated for three hours at 37°C in 5% CO_2_. Post incubation, the bound neutral red dye was eluted from the cells using an ethanol-acetic acid de-staining solution. The optical density of the released dye was measured at 540 nm using a microplate reader Multiskan SkyHigh Microplate Spectrophotometer (Thermo Fisher Scientific Inc.). Cell viability was determined as a relative percentage of untreated control cells. The values determined using this assay were used to determine the IC50 for our further experiments.

Evaluation of the influence of GFC on the migratory capability of methylprednisolone-treated cells using the wound healing/scratch assay

The migration of cells to the injured area is an essential process in wound healing. The in vitro wound healing scratch assay has been widely used to demonstrate cell migration [23]. Here, we evaluated the regenerative capacity of GFC by assessing cell migration in methylprednisolone-treated ACL-derived stromal cells and chondrocytes.

The cells were seeded in 48-well tissue culture plates (TPP) and cultured to the confluence at 37˚C with 5% CO_2_. A small linear scratch was created in the confluent monolayer in each well with a 200 µl pipette tip. Cells were rinsed twice with sterile 1X PBS (CellClone) to remove any cellular debris before the addition of the test combinations.

Methylprednisolone was used at a concentration of 350 μg/ml based on the IC50 values determined by the NRU assay. Moreover, since 20% and 50% GFC showed the same viability (>90%) by the NRU assay, 20% GFC was used for this assay.

In the first set of combinations, the ACL-derived stromal cells and chondrocytes were treated with only methylprednisolone (C1) and a combination of methylprednisolone and GFC (C2) to determine if simultaneous GFC usage mitigates methylprednisolone mediated cell toxicity (Table 2).

**Table 2:** Wound healing scratch assay combinations GFC: growth factor concentrate, HSA: human serum albumin

Study groups	Test combinations	Media replacement post six hours up to 48 hours
C1	Treatment with methylprednisolone (350 μg/ml) for 48 hours	N/A
C2	Treatment with methylprednisolone (350 μg/ml) + GFC (20%) for 48 hours	N/A
T1	Treatment with methylprednisolone (350 μg/ml) for six hours	Complete media w/ 20% GFC
T2	Treatment with a combination of methylprednisolone (350 μg/ml) + 20% GFC for six hours	Complete media w/ 20% GFC
T3	Treatment with methylprednisolone (350 μg/ml) for six hours	Complete media w/ 20% HSA
T4	Treatment with a combination of methylprednisolone (350 μg/ml) + 20% GFC for six hours	Complete media w/ 20% HSA

Next, to assess the cytoprotective nature of GFC and validate its contribution to wound closure, parallel experiments were performed in two groups, where the scratched cell layers were treated with only methylprednisolone and a combination of methylprednisolone + GFC for six hours. After six hours, in one group, the treatment media were replaced with media containing 20% GFC (T1, T2), while in another group, the treatment media were replaced with 20% human serum albumin (HSA) (T3, T4) (Table 2). The use of HSA was done in order to validate the specificity of GFC in cytoprotective effects.

The extent of wound healing in all combinations was determined by counting the number of cells migrating into the denuded area at the end of 48 hours and compared with the zero-hour time point. Imaging was performed using an Olympus CKX53SF inverted microscope, equipped with Magcam DC 5 camera and MagVision 4.6 software. The area of wound healing was analyzed using the GNU Image Manipulation Program- GIMP 2.10.24 (revision 2).

Evaluation of the ability of GFC to rescue the morphological changes induced by methylprednisolone

Cell morphological changes due to methylprednisolone treatment and the effects of GFC in cytoprotection were studied by treating ACL-derived stromal cells and chondrocytes with methylprednisolone (350 μg/ml) and a combination of methylprednisolone (350 μg/ml) and GFC (20% v/v) for six hours. After six hours, the treatment media were replaced with media containing 20% GFC (v/v). At six-hour and 24-hour time points, cell morphology was assessed by standard H&E staining; the collagen I pattern was analyzed by immunostaining.

The ultrastructure of cells treated as above with methylprednisolone and GFC was assessed using transmission electron microscopy (TEM). For ultrastructure analysis, cells at six-hour, 24-hour, and 72-hour time points were fixed with 3% glutaraldehyde (Ted Pella, United States) and post-fixed in 1% aqueous osmium tetroxide (Ted Pella), followed by en block staining in 2% uranyl acetate. The cells were then subsequently dehydrated in an ascending alcohol series, followed by infiltration and finally embedding in Araldite 502 resin (Ted Pella). The polymerized resin blocks were ultrathin sectioned at 70 nm and contrasted with lead citrate. Images were captured using a transmission electron microscope, JEM 1400 Plus (JEOL, Japan) at 120 kV.

Statistical analysis

Statistical analysis was performed using GraphPad software version 10.1.2 (United States). Statistical significance was tested using a two-way analysis of variance (ANOVA) with Tukey’s multiple comparison tests, where p < 0.05 was considered statistically significant.

## Results

Isolation and characterization of primary ACL-derived stromal cells

**Figure 1:**
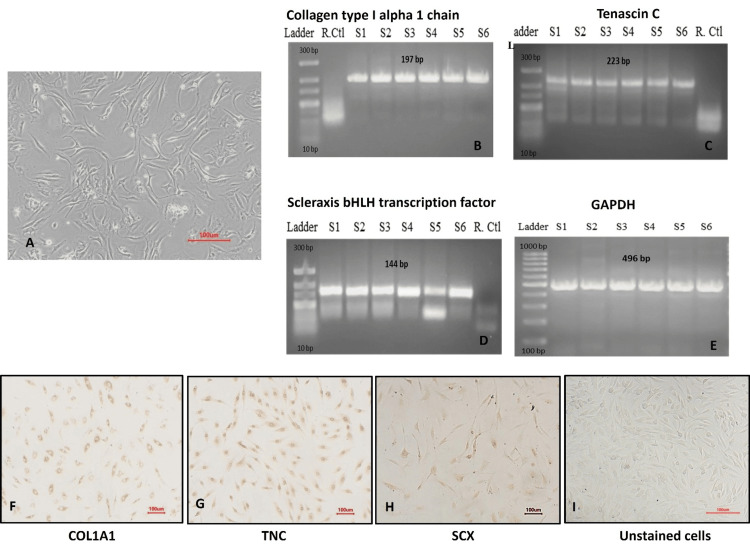
Characterization of primary ACL-derived stromal cells (A) Spindle-shaped morphology; (B-E) positive expression of cell-specific markers COL1A1, tenascin C, scleraxis A, and GAPDH by RT-PCR (n = 6); (F-H) immunocytochemistry micrographs of COL1A1, tenascin C, and scleraxis A with (I) unstained cells (10X; scale= 100 µm). ACL: anterior cruciate ligament, GAPDH: glyceraldehyde 3-phosphate dehydrogenase, RT-PCR: reverse transcription polymerase chain reaction

ACL-derived stromal cells, isolated from ACL tissue fragments, exhibited a fibroblastic morphology, as illustrated in Figure 1A. ACL stromal cells showed positive expression of CD44 (>85%), CD90 (>95%), and CD105 (>85%) and absence (<5%) of the lymphocyte cell marker CD45 and the endothelial cell marker CD31, as documented in Table 3.

**Table 3:** Anterior cruciate ligament (ACL)-derived stromal cell marker expression

Sample	ACL-derived stromal cell marker expression			Negative marker expression	
	CD44	CD90	CD105	CD31	CD45
1	84.9%	99.5%	82.4%	0.29%	0.13%
2	88.5%	99.5%	95.4%	0.1%	0.13%
3	99.2%	99.6%	97.1%	0.04%	0.09%
4	99.9%	100%	99.1%	0.09%	0.08%
5	83.5%	99.8%	97.3%	1.06%	0.68%
6	99.4%	99.6%	97.1%	0.14%	0.29%

Reverse transcriptase PCR revealed a positive expression of COL1A1, tenascin C, and scleraxis A. The GAPDH housekeeping gene was used as the internal control (Figure 1B-1E).

Further validation of the ACL-derived stromal cell phenotype was performed by immunocytochemistry (ICC) analysis using collagen type I, scleraxis A, and tenascin C markers. Brighter stain expressions of collagen type I and scleraxis A were observed, while tenascin C expression appeared dim (Figure 1F-1I).

Collectively, the stromal cells isolated from ACL tissue expressed characteristic lineage-specific markers, as evidenced by the analyses of cell surface markers, RNA expression, and immunocytochemistry.

Isolation and characterization of primary chondrocytes

**Figure 2:**
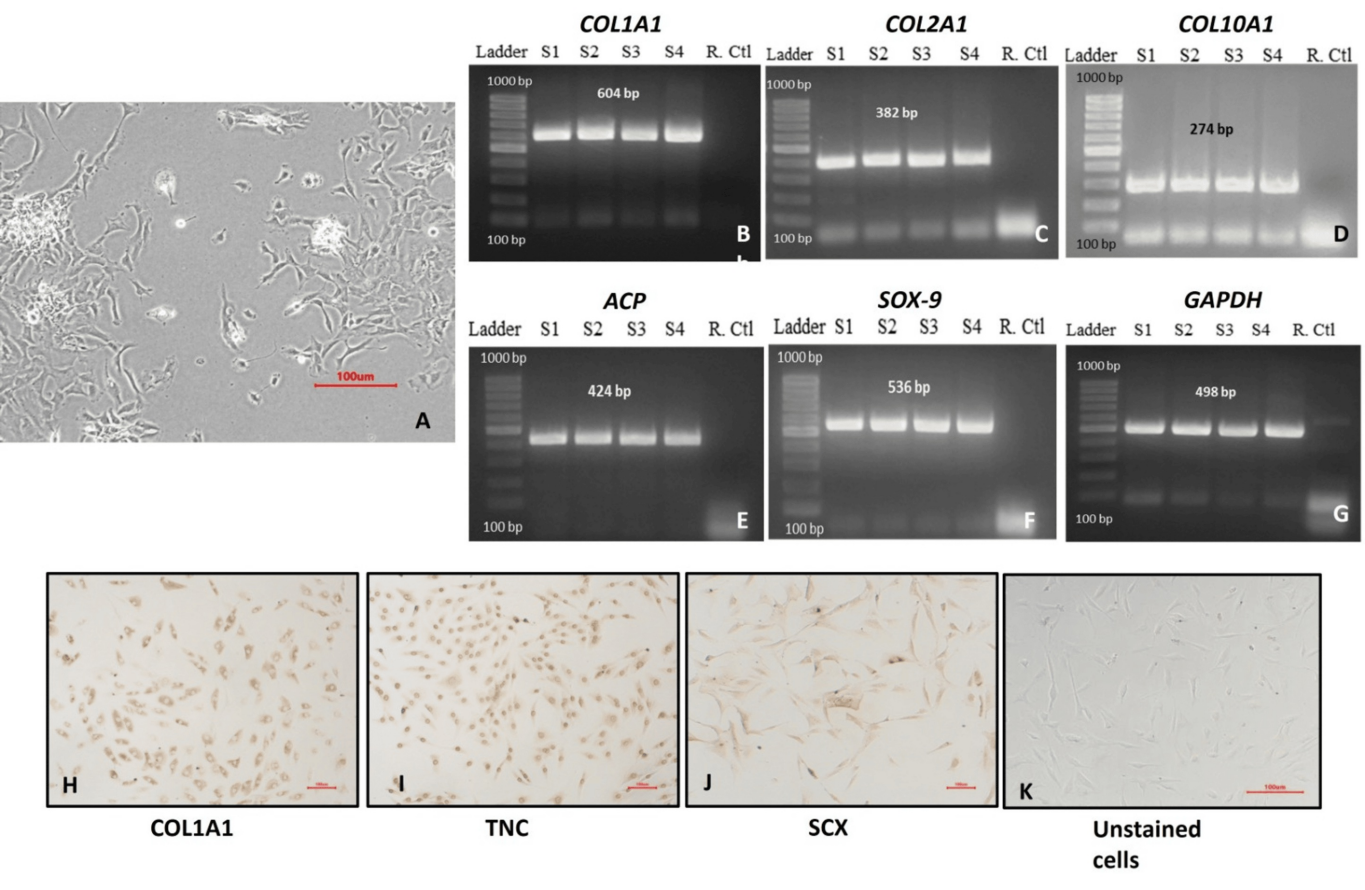
Characterization of primary chondrocytes (A) Polygonal morphology; (B-G) positive expression of cell-specific markers COL1A1, COL2A1, ACP, SOX-9, COL X, COL10A1, and GAPDH by RT-PCR (n = 4); (H-J) immunocytochemistry micrographs of COL1A1, tenascin C, and scleraxis A with (K) unstained cells (10X; scale = 100 µm). COL1A1: collagen type I alpha 1 chain, COL2A1: collagen type II alpha 1 chain, ACP: acid phosphatase, SOX-9: SRY-Box Transcription Factor 9 [SRY: Sex-determining region Y], COL X: collagen type X, COL10A1: Collagen Type X Alpha 1 chain, GAPDH: glyceraldehyde 3-phosphate dehydrogenase, RT-PCR: reverse transcription polymerase chain reaction

Cells isolated from cartilage had a polygonal structure and exhibited spindle/fibroblast-like morphology consistent with chondrocytes (Figure 2A).

The cells, phenotyped via flow cytometry, exhibited the surface expression of chondrocyte markers CD44 (>95%), CD151 (>95%), CD49c (>95%), and HLA-ABC (>95%). In addition, they displayed (>95%) positivity for CD73, CD90, and CD105, which are expressed by mesenchymal stromal cells. A small population of chondrocyte cells (about <10%) expressed hematopoietic lineage cell marker CD45 and immune response marker HLA-DR (Table 4).

**Table 4:** Chondrocyte marker expression

Sample	Mesenchymal stromal cell markers			Chondrocyte marker	Chondrocyte potency markers		HLA class I marker	Negative markers	
	CD73	CD90	CD105	CD44	CD151	CD49c	HLA- ABC	CD45	HLA-DR
1	98.5%	99.8%	97.7%	99.3%	99.7%	98.0%	97.4%	7.17 %	3.97 %
2	97.6%	99.0%	98.1%	98.3%	98.3%	95.8%	97.6%	2.96 %	1.72 %
3	98.6%	99.5%	97.5%	98.7%	99.5%	97.3%	99.6%	6.48 %	4.70 %
4	99.2%	99.7%	95.4%	98.2%	98.6%	97.9%	98.7%	7.91 %	6.60 %

The chondrocyte lineage was further validated through RNA-based reverse transcriptase PCR analysis, revealing positive expression of chondrocyte-specific genes including COL1A1, COL2A1, ACP, SOX-9, COL X, and COL10A1. GAPDH housekeeping gene was used as the internal control (Figure 2B-2G).

The chondrocytes were positive for COL1A1, tenascin C, and scleraxis A markers assessed by ICC. Chondrocytes exhibited particularly enhanced tenascin C expression compared to ACL-derived stromal cells (Figure 2H-2K).

Collectively, primary cartilage cells isolated from cartilage tissue consistently expressed lineage markers, as evidenced through analyses of cell surface expression, RNA, and immunocytochemistry.

Multipotent differentiation of ACL-derived stromal cells

**Figure 3:**
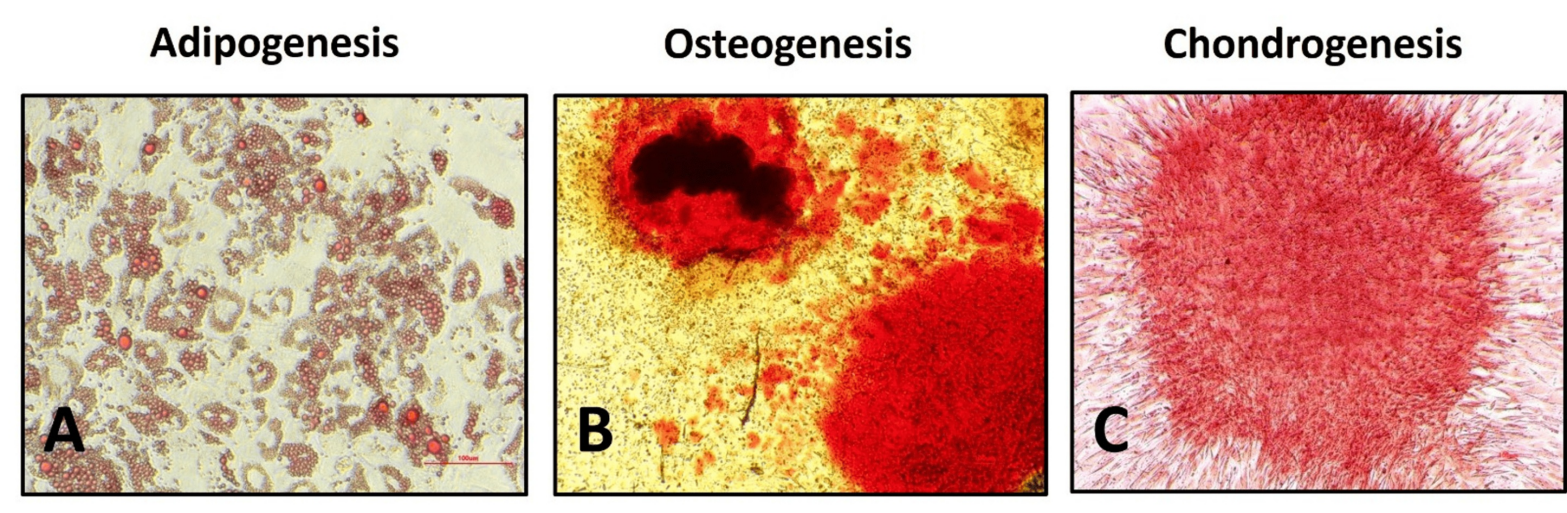
Tri-lineage differentiation potential of ACL-derived stromal cells Anterior cruciate ligament (ACL)-derived cells differentiated into (A) adipocytes (B) osteoblasts and (C) chondrocytes (n = 4) (4X; scale = 100 μm).

ACL-derived stromal cells isolated in this study differentiated into adipogenic, osteogenic lineages identified using oil red O and alizarin red staining, respectively, at day 21 (Figure 3A, 3B). ACL-derived stromal cells differentiated into chondrogenic lineage were identified using safranin O staining of micromass pellets between days 7 and 10 (Figure 3C). The control cells did not show any positivity for the adipogenic, osteogenic, and chondrogenic lineages

Methylprednisolone-induced cytotoxicity reversed by GFC

The effects of GFC and methylprednisolone on cell viability were assessed using the lysosome-based neutral red uptake (NRU) assay. GFC, prepared using the Ossinext^TM^ GFC kit, had growth factors TGF-β (60618.6 pg/mL), PDGF (2538.26 pg/mL), VEGF (335.79 pg/mL), EGF (391.84 pg/mL), and IL-1ra (570.12 pg/mL) assessed using ELISA.

**Figure 4:**
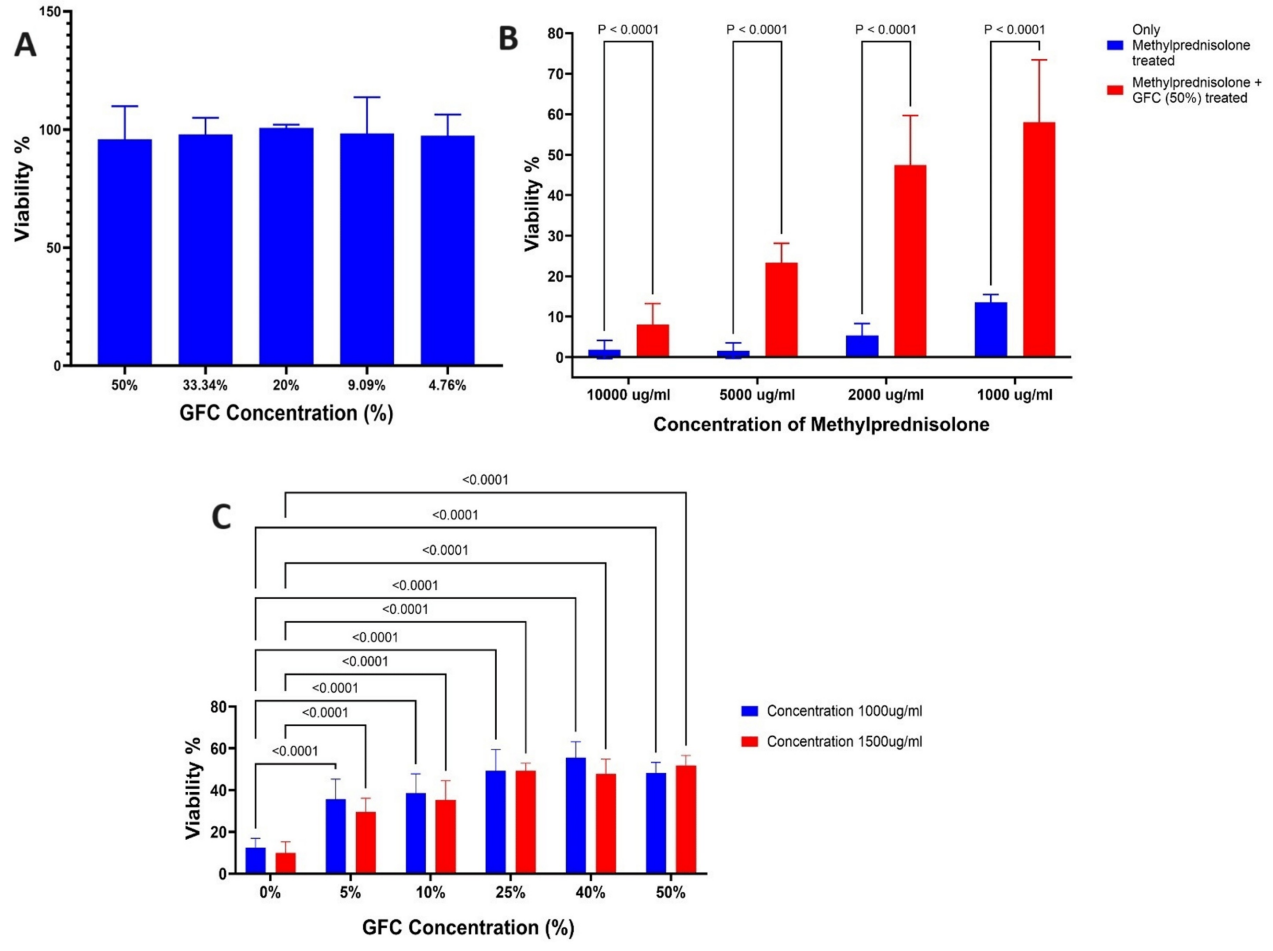
Effect of methylprednisolone and GFC on ACL-derived stromal cell viability (A) >90% viability at different GFC concentrations. (B) Methylprednisolone + GFC (50%) significantly boosts viability (P < 0.0001) vs. methylprednisolone alone. (C) 1000 μg/ml and 1500 μg/ml of methylprednisolone with various GFC concentrations show significantly improved viability (P < 0.0001) vs. methylprednisolone alone (n = 4). GFC: growth factor concentrate, ACL: anterior cruciate ligament

**Figure 5:**
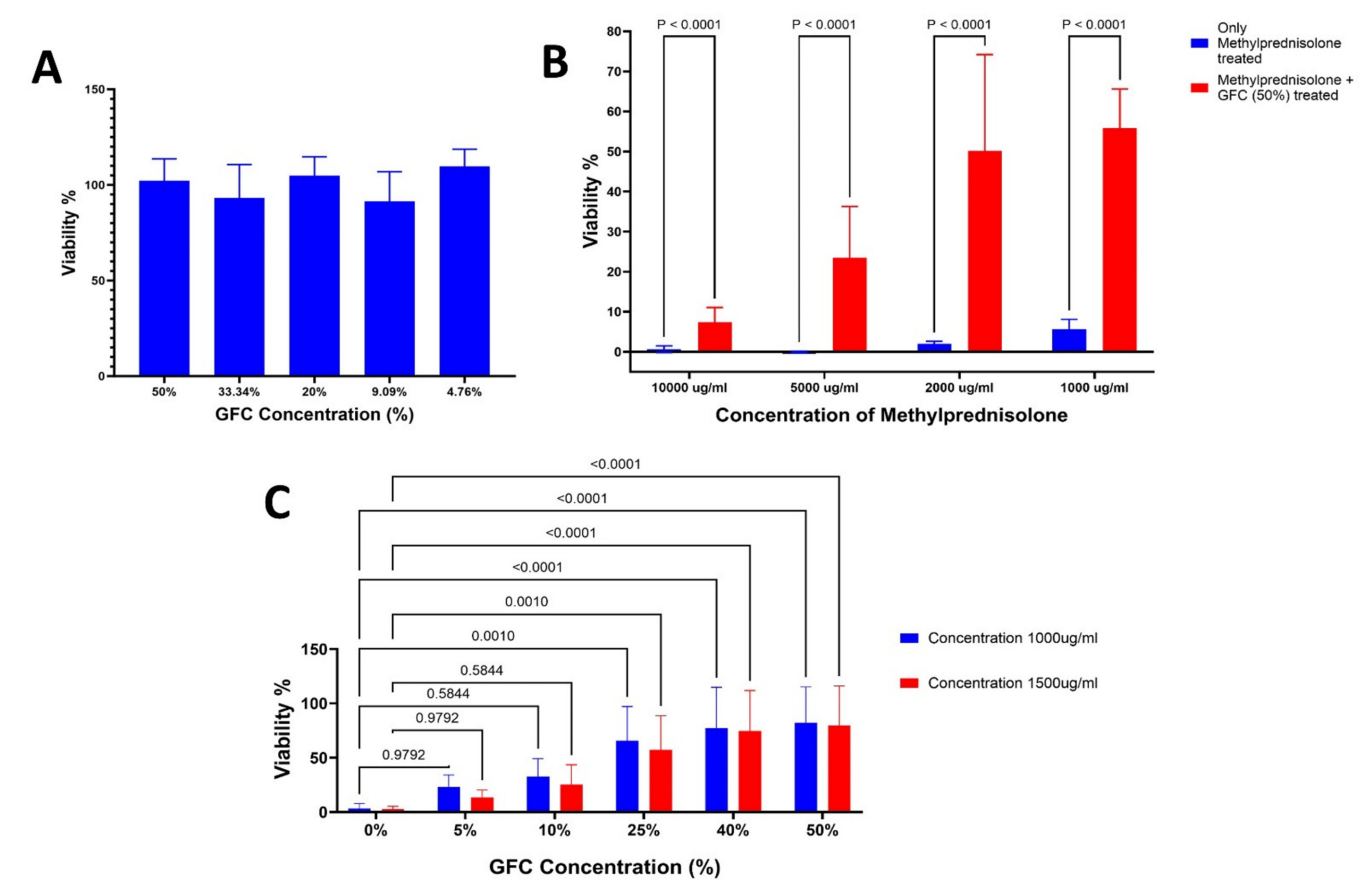
Effect of methylprednisolone and GFC on chondrocyte viability (A) >90% viability at different GFC concentrations. (B) Methylprednisolone + GFC (50%) significantly boosts viability (P < 0.001) vs. methylprednisolone alone. (C) 1000 μg/ml and 1500 μg/ml of methylprednisolone with various GFC concentrations show significantly improved viability (P < 0.001) vs. methylprednisolone alone (n = 4). GFC: growth factor concentrate

Viabilities of ACL-derived stromal cells and chondrocytes remained unaltered with increasing concentrations of GFC, with both cell types retaining >90% cell viability (Figures 4A, 5A).

Increasing concentrations of methylprednisolone decreased the cell viability in both ACL-derived stromal cells and chondrocytes and the IC50 was found to be 993.1µg/ml.

Experiments were performed to demonstrate the cytoprotective effects of GFC by measuring cell viability upon the addition of 50% GFC concomitantly with methylprednisolone. Statistically significant increases in cell viabilities were observed in both ACL-derived stromal cells (P < 0.001 for all the concentrations tested) and chondrocytes (P < 0.001 for all the concentrations tested) as compared to methylprednisolone-treated cells (Figures 4B, 5B).

Based on the IC50 value, additional experiments were performed using two increasing concentrations of methylprednisolone - 1000 µg/ml and 1500 µg/ml. GFC was added concurrently at different concentrations ranging from 0% to 50%. This experiment was performed to explore whether GFC has any cytoprotective effects beyond the IC50 of methylprednisolone determined in our study.

Increasing concentrations of GFC significantly increased the cell viabilities of ACL-derived stromal cells treated with 1000 µg/ml and 1500 µg/ml of methylprednisolone as compared to methylprednisolone treated cells (P < 0.0001 for all the GFC concentrations tested) (Figure 4C). Similar observations were found in chondrocytes (Figure 5C). However, the statistical significance was observed only between 25% and 50% of GFC (P < 0.0001)

Collectively, these data suggest that the concomitant addition of GFC with methylprednisolone increases cell viability as compared to only methylprednisolone-treated cells. Interestingly, GFC was able to improve cell viability beyond the IC50 values of methylprednisolone-treated ACL-derived stromal cells and chondrocytes.

​GFC treatment induces migration and proliferation in methylprednisolone induced cells

In this study, we explored the potential of GFC in promoting the regeneration of cytotoxic events induced by methylprednisolone treatment in cells derived from connective tissues of ACL-derived stromal cells and chondrocytes using scratch wound healing assay. Methylprednisolone was used at a concentration of 350 μg/mL, as IC50 concentrations caused significant cell death, limiting the duration of the experiment.

**Figure 6:**
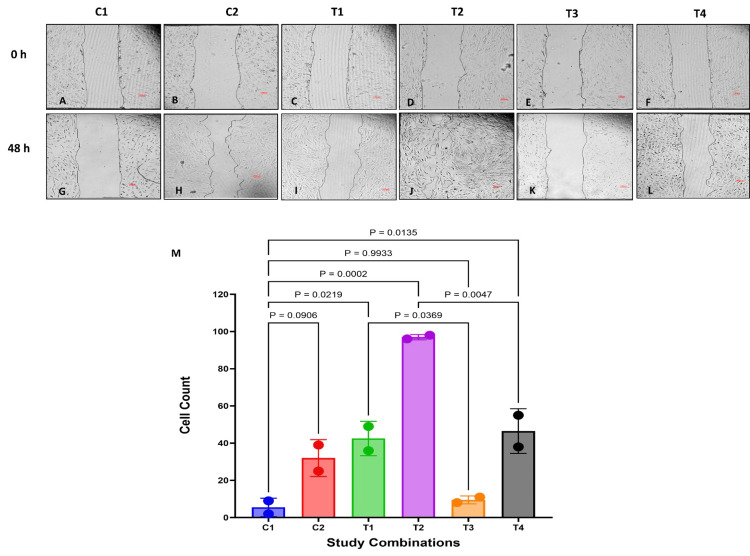
Effect of GFC on cell migration in methylprednisolone-treated ACL cells (A-L) Cell migration at 48 hours (scale: 100 µm); (M) images were quantitatively analyzed (n = 2). P < 0.05 is considered significant. GFC: growth factor concentrate, ACL: anterior cruciate ligament

**Figure 7:**
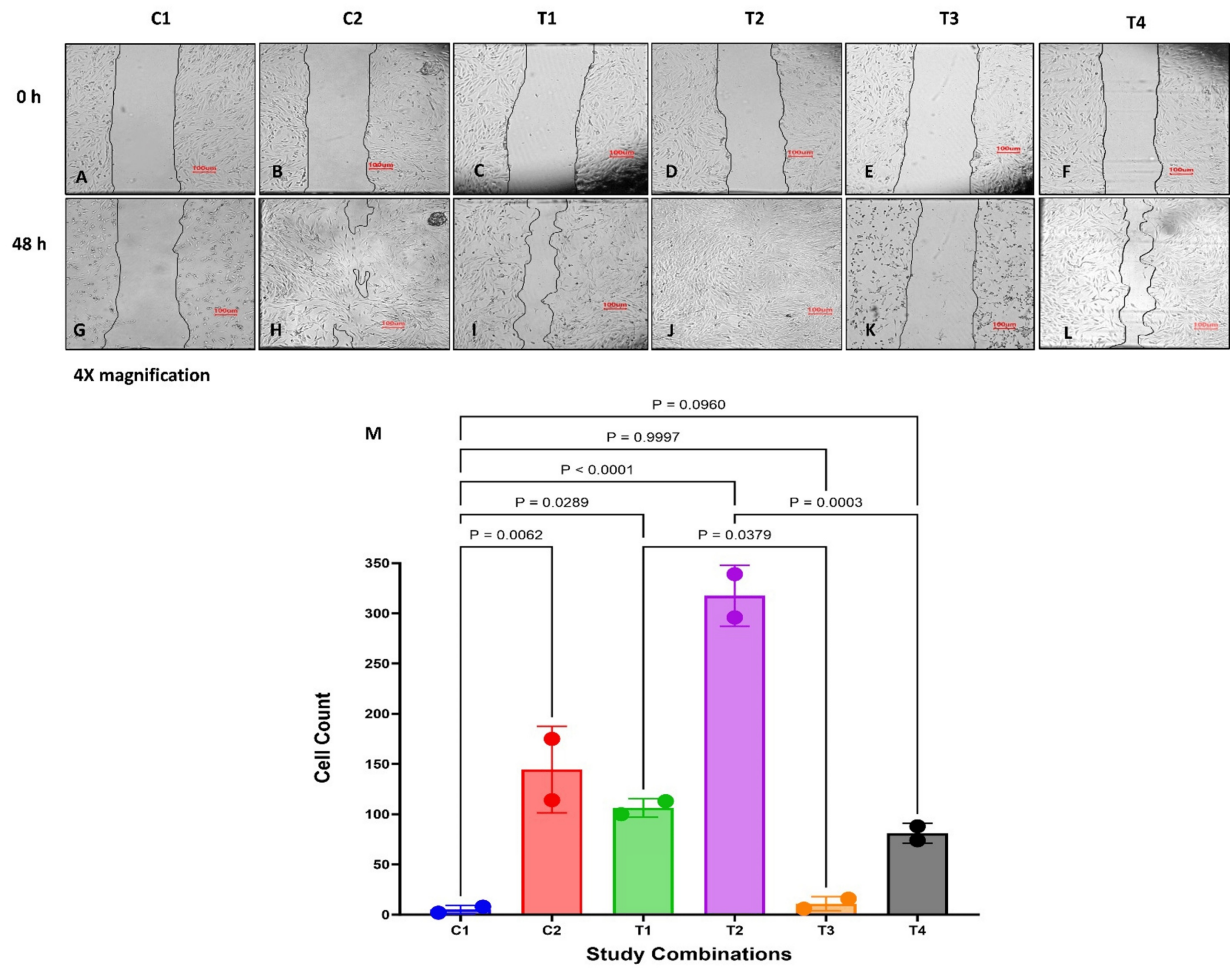
Effect of GFC on cell migration in methylprednisolone-treated chondrocytes (A-L) Cell migration at 48 hours (scale: 100 µm); (M) images were quantitatively analyzed (n = 2). P < 0.05 is considered significant. GFC: growth factor concentrate

ACL-derived stromal cells and chondrocytes treated with methylprednisolone (C1) showed no substantial cell proliferation and cell migration at the end of 48 hours (Figure 6A, 6G, 7A, 7G).

In ACL-derived stromal cells, the concomitant addition of 20% GFC with methylprednisolone (C2) showed a non-significant improvement in cell proliferation compared to cells treated only with methylprednisolone (P = 0.09) (Figure 6B, 6H). Cells treated with only methylprednisolone for six hours, when further treated with 20% GFC (T1), resulted in a significant enhancement in cell proliferation compared to cells treated with only methylprednisolone (P = 0.02) as observed by the decrease in wound gap at 48 hours (Figure 6C, 6I). Notably, concomitant treatment of methylprednisolone with 20% GFC for six hours, followed by media replacement with 20% GFC (T2), led to significant enhancement in cell proliferation (P = 0.0002) (Figure 6D, 6J).

To assess the specificity of GFC in regenerating corticosteroid-injured cells, experiments were conducted using 20% HSA. Cells treated with only methylprednisolone for six hours, followed by treatment with 20% HSA (T3) did not exhibit improved cell proliferation (P = 0.99) (Figure 6E, 6K). However, concomitant treatment of methylprednisolone with 20% GFC for six hours, followed by media replacement with 20% HSA (T4), significantly improved cell proliferation (P = 0.01) (Figure 6F, 6L). The degree of improvement in cell proliferation was however lesser when compared to cells treated with 20% GFC (Figure 6J, 6L).

In chondrocytes, similar observations were noted. Simultaneous addition of 20% GFC with methylprednisolone (C2) significantly improved cell proliferation compared to cells treated only with methylprednisolone at 48 hours (P = 0.006) (Figure 7B, 7H). Treatment of six-hour methylprednisolone-exposed cells with 20% GFC (T1) also led to a significant enhancement in cell proliferation compared to cells treated with only methylprednisolone (P = 0.02) (Figure 7C, 7I). Chondrocytes initially treated with GFC in combination with methylprednisolone, followed by further treatment with GFC (T2), showed remarkable cell proliferation and migration, with the scratch area almost completely covered at the end of 48 hours (P < 0.001) (Figure 7D, 7J).

To corroborate the specificity of GFC in regenerating corticosteroid-injured cells, experiments were conducted using 20% HSA, akin to those performed with ACL-derived stromal cells. Methylprednisolone-treated chondrocytes treated with 20% HSA (T3) did not exhibit improved cell proliferation (P = 0.99) (Figure 7E, 7K). Cell treated concomitantly with methylprednisolone and 20% GFC for six hours, followed by media replacement with 20% HSA (T4), showed non-significant improvement in cell proliferation (P = 0.09) (Figure 7F, 7L). As observed in ACL-derived stromal cells, the improvement in chondrocyte cell proliferation was also lesser compared to recovery with GFC (Figure 7J, 7L).

The results from scratch-wound assay indicate that GFC has the ability to regenerate cells from methylprednisolone-induced cytotoxicity evidenced by proliferation and migration, which aided in closing the wound areas.

GFC treatment reverses the methylprednisolone-induced cell morphology changes

We aimed to investigate whether defects in cell morphology and ultrastructure are observed in cells treated with methylprednisolone and whether the addition of GFC could ameliorate these events.

**Figure 8:**
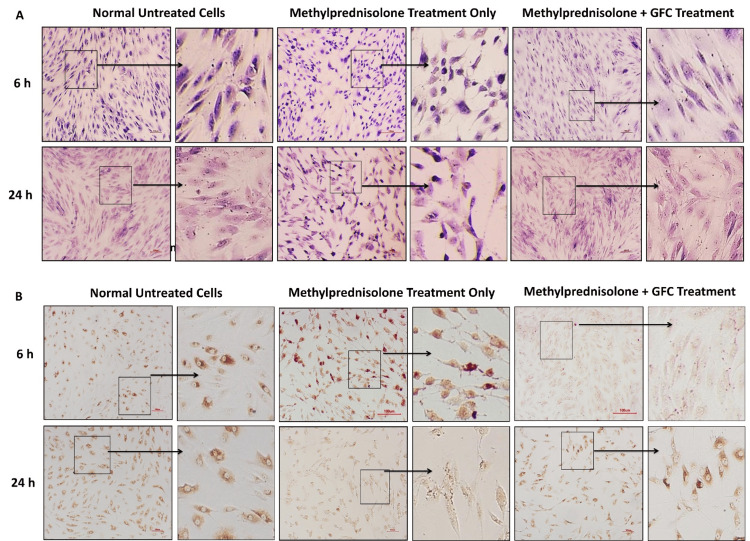
Effects of GFC on cell morphology and collagen structure in methylprednisolone-treated ACL cells (A) H&E staining, (B) collagen immunostaining of treated ACL cells. Methylprednisolone-treated cells show altered morphology and collagen patterns, while those treated with GFC concurrently and subsequently resemble untreated cells (n = 4) (10X; scale = 100 µm). GFC: growth factor concentrate, ACL: anterior cruciate ligament

The methylprednisolone treatment induced differences in cell size compared to untreated cells, as revealed by H&E staining in both ACL-derived stromal cells and chondrocytes (Figure 8A, 9A). Cells treated concomitantly with methylprednisolone and 20% GFC did not exhibit these altered cell morphologies (Figure 8A, 9A). The morphologies of GFC-treated ACL-derived stromal cells and chondrocytes were similar to untreated control cells, suggesting the role of GFC in improving methylprednisolone-induced altered cell morphologies.

**Figure 9:**
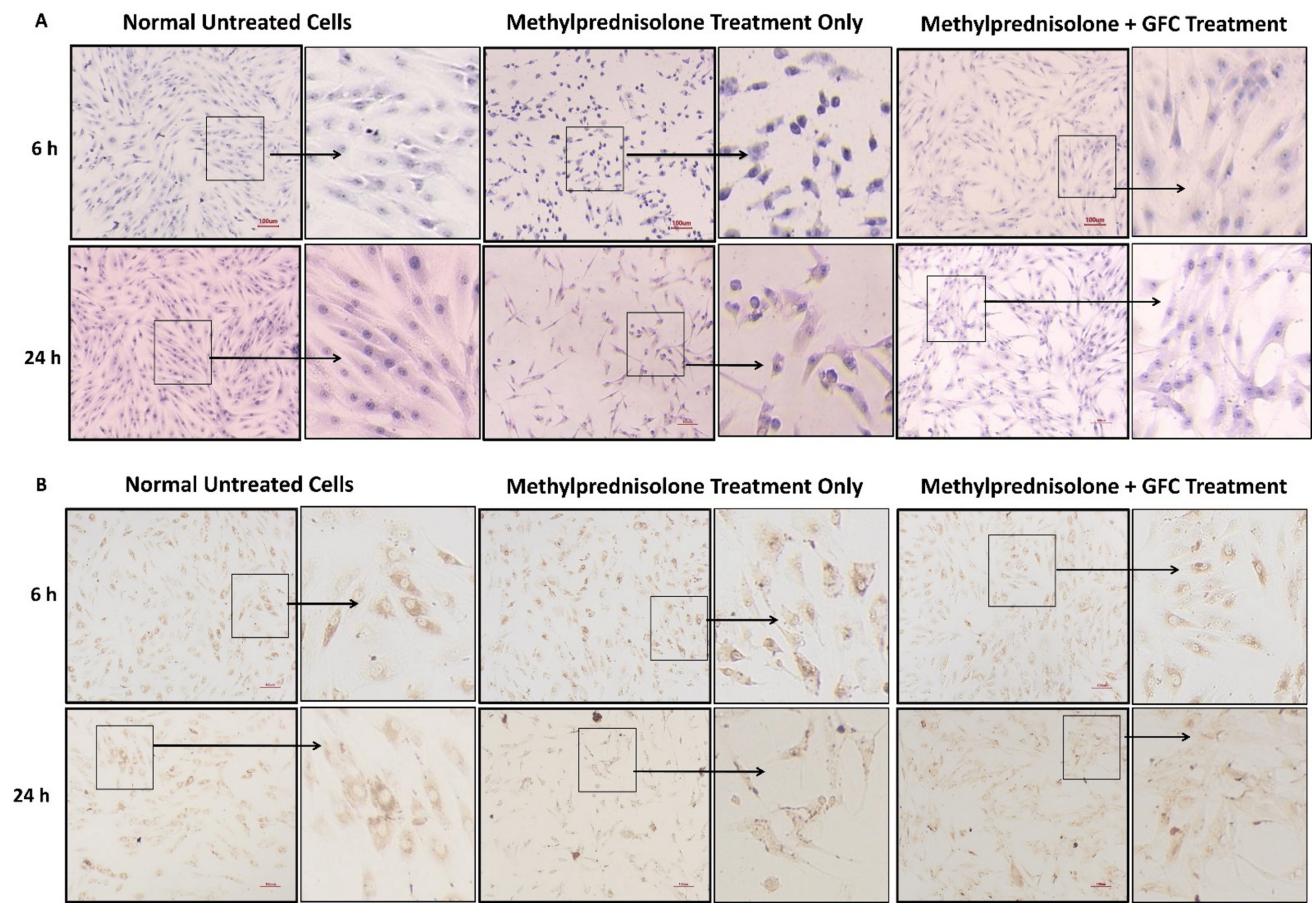
Effects of GFC on cell morphology and collagen structure in methylprednisolone-treated chondrocytes (A) H&E staining, (B) collagen immunostaining of treated chondrocytes. Methylprednisolone-treated cells show altered morphology and collagen patterns, while those treated with GFC concurrently and subsequently resemble untreated cells (n = 4) (10X; scale = 100 µm). GFC: growth factor concentrate

We assessed the expression of extracellular matrix protein collagen I in ACL-derived stromal cells and chondrocytes. In untreated ACL-derived stromal cells, collagen I staining exhibited a uniform distribution in the cytoplasm. However, in cells treated with methylprednisolone, uneven collagen I staining was observed, with localization toward the nucleus, consistent with the altered cell morphology observed in H&E staining. Concomitant addition of 20% GFC along with methylprednisolone resulted in collagen I staining patterns similar to those observed in untreated cells. Chondrocytes exhibited similar results to ACL-derived stromal cells (Figure 8B, 9B).

The ultra-structural changes in ACL-derived stromal cells and chondrocytes following methylprednisolone and GFC treatment were examined using TEM.

**Figure 10:**
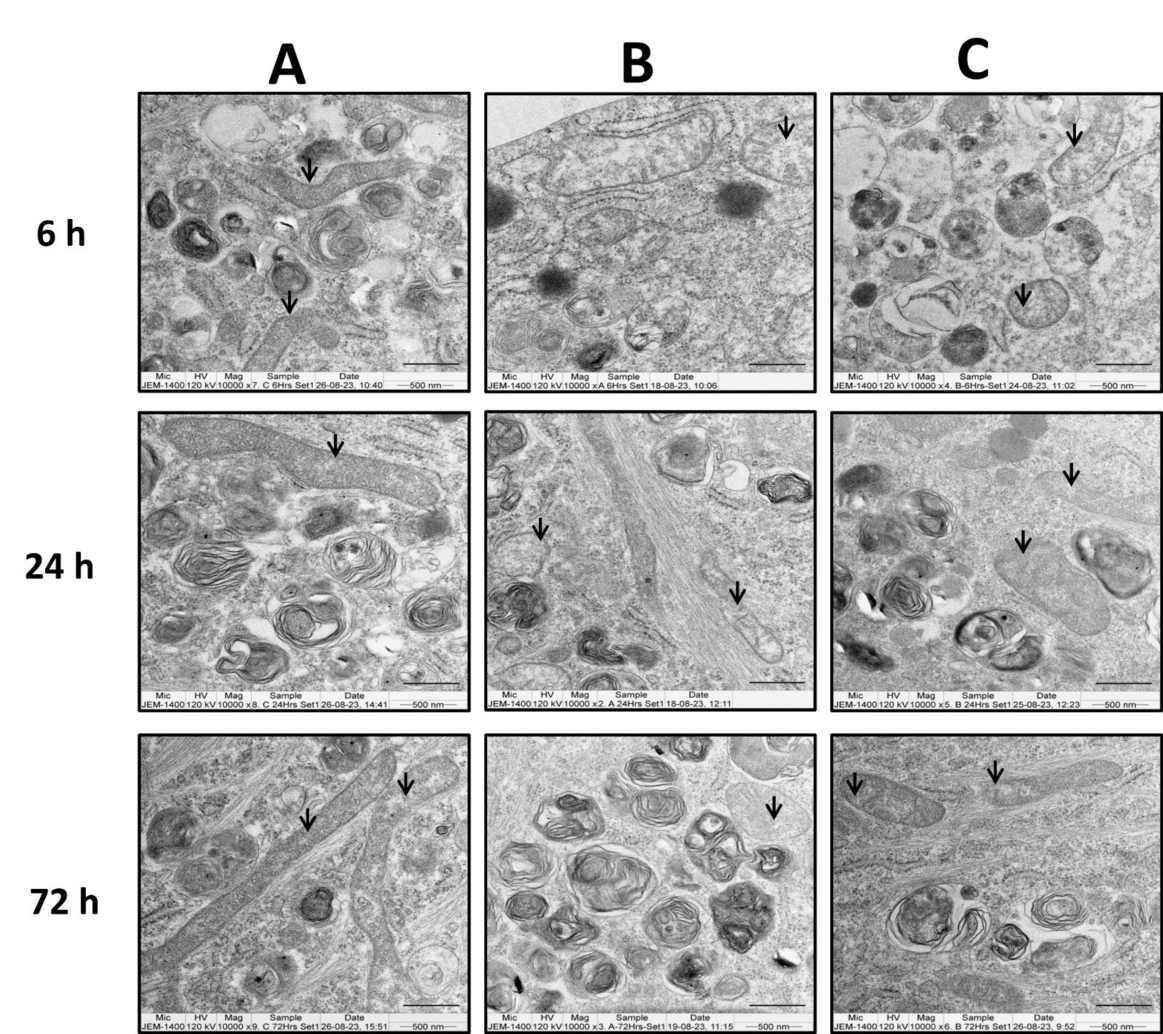
GFC effects on methylprednisolone-induced organelle damage in ACL-derived stromal cells assessed by TEM at 6, 24, and 72 hours. Control cells (A) had normal mitochondrial morphology. Mitochondrial defects observed in methylprednisolone-treated cells (B). Methylprednisolone + GFC treatment (C) had reversed morphology (n = 2). Arrows indicate mitochondria. GFC: growth factor concentrate, ACL: anterior cruciate ligament, TEM: transmission electron microscopy

**Figure 11:**
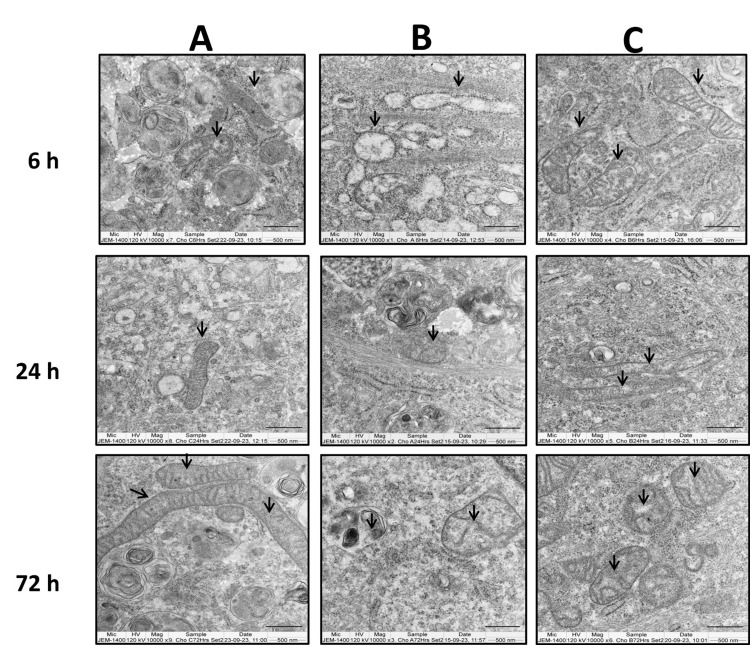
GFC effects on methylprednisolone-induced organelle damage in chondrocytes assessed by TEM at at six, 24, and 72 hours Control cells (A) had normal mitochondrial morphology. Mitochondrial defects observed in methylprednisolone-treated cells (B). Methylprednisolone + GFC treatment (C) had reversed morphology (n = 2). Arrows indicate mitochondria. GFC: growth factor concentrate, TEM: transmission electron microscopy

Both cell types exhibited loss of cristae in the mitochondria after treatment with methylprednisolone alone (Figures 10B, 11B), as observed at the six-hour time point as compared to their untreated controls (Figures 10A, 10B). However, concurrent treatment with 20% GFC and methylprednisolone showed mitochondria with normal cristae, similar to untreated control cells (Figures 10C, 11C) at six hours.

Following the six-hour methylprednisolone treatment, the addition of 20% GFC led to a partial reversal in the structure of mitochondrial cristae at 24 and 72 hours in both ACL-derived stromal cells and chondrocytes (Figure 10C, 11C).

**Figure 12:**
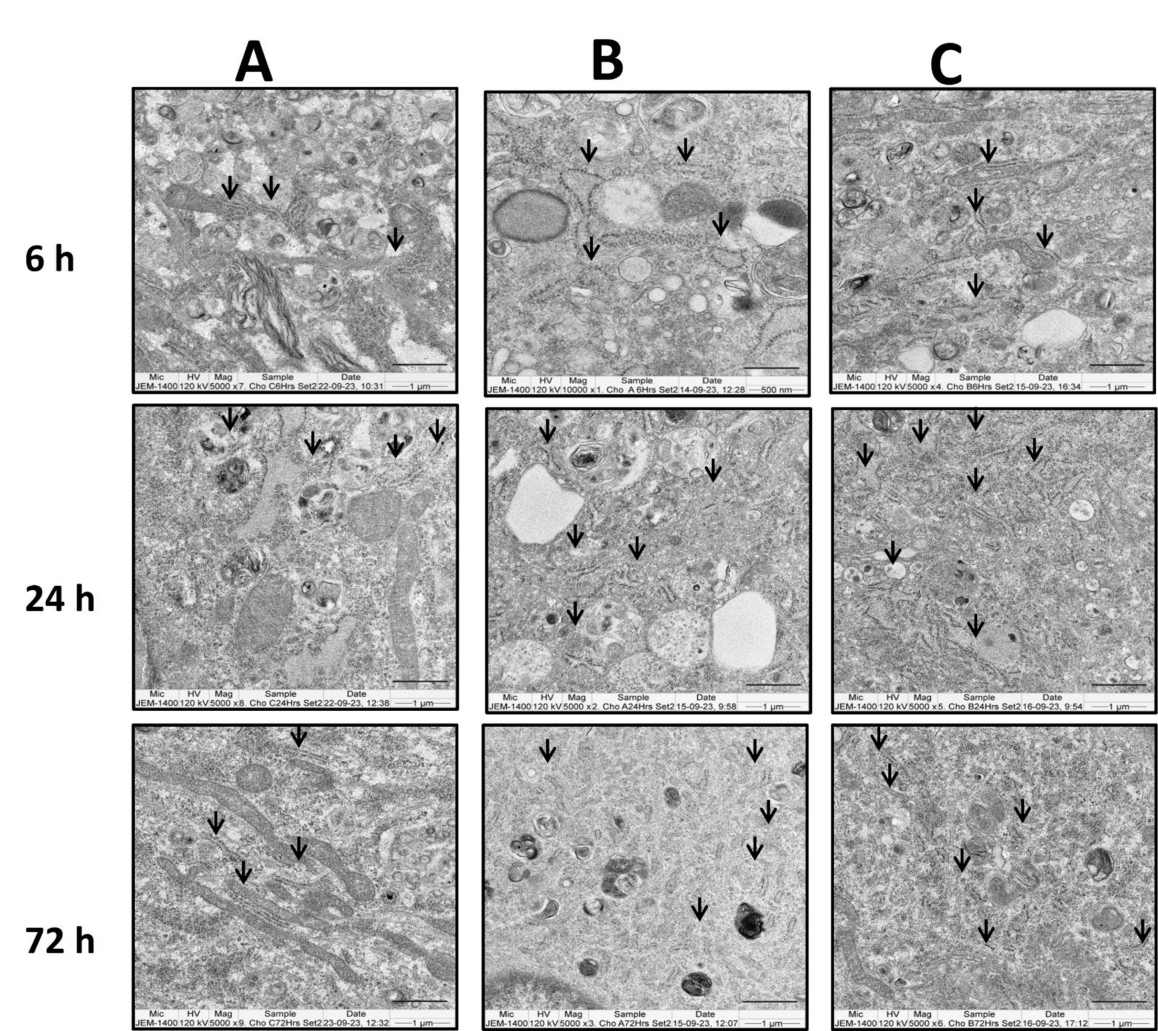
GFC effects on methylprednisolone-induced organelle damage in chondrocytes assessed by TEM at six, 24, and 72 hours. Control cells (A) had normal ribosomal morphology. Ribosomal swelling observed in methylprednisolone-treated cells (B). Methylprednisolone + GFC treatment (C) had reversed morphology (n = 2). Arrows indicate ribosomes. GFC: growth factor concentrate, TEM: transmission electron microscopy

In chondrocytes treated with methylprednisolone, ribosomal swelling was observed (Figure 12B), which was not observed in cells treated simultaneously with methylprednisolone and GFC and the untreated control cells at the six-hour time point (Figure 12A, 12C). When methylprednisolone-treated cells were subsequently treated with GFC after six hours, partial recovery of the ribosomal structures was noted (Figure 12B, 12C).

Taken together, these findings suggest that GFC has the potential to mitigate cell damage induced by methylprednisolone when used in combination with the corticosteroid.

## Discussion

This study examines the efficacy of Wockhardt Ossinext^TM^ kit-derived GFC in ameliorating corticosteroid-induced cytotoxicity in ACL-derived stromal cells and connective tissue-derived chondrocytes in vitro. Corticosteroid injections are commonly used for joint pain relief but are known to adversely affect tendon, ligament, and cartilage homeostasis as documented in previous in vitro and in vivo studies [5,6,24,25].

PRP and its derivatives, including GFC produced by platelet clotting resulting in acellular serum, are recognized for their proliferative, anabolic, and cytoprotective properties against corticosteroids in ACL cells, tenocytes, and chondrocytes in experimental and clinical settings [12,13,26].

The ACL stromal cells and chondrocytes used in this study were phenotyped using flowcytometry, RT-PCR, and immunocytochemistry techniques. ACL stromal cells exhibited fibroblast morphology along with multilineage differentiation potential and expressed CD44, CD90, CD105, scleraxis, collagen I, and tenascin C, which were reported to be expressed either by stem cells, connective tissue fibroblasts, or chondrocytes [16-21].

The cartilage-derived cells exhibited fibroblastic morphology along with the cell surface expression of CD44, CD151, and CD49c and gene expressions of COL1A1, COL2A1, ACP, SOX-9, and COL10A1, which were reported to be expressed by chondrocytes [18,19,20]. Both ACL-derived stromal cells and chondrocytes had a negative expression of hematopoietic markers authenticating their lineages.

Initially, we assessed the effects of methylprednisolone on cell viability using the NRU assay. Methylprednisolone-treated ACL stromal cells and chondrocytes exhibited reduced cell viability. In line, these cells also exhibited cell migration defects in vitro scratch wound healing assay. Methylprednisolone-treated cells exhibited altered cell morphology evinced from H and E staining and TEM data and altered ECM protein collagen-I expression. Hence, the addition of methylprednisolone during pain management in connective tissue injuries causes cytotoxicity along with cell damage. Based on our data, the concomitant addition of GFC along with methylprednisolone showed statistically significant improvements in cell viability, cell proliferation along with cell morphology, recovery of collagen I staining defects, and maintenance of cell organelle integrity were observed. These findings were similar to studies published earlier where PRP was shown to induce cytoprotective effects [12,27].

Similar to our observations of changes in organelle integrity like swollen ribosomes, mitochondrial cristae defects in both methylprednisolone-treated ACL-derived stromal cells and chondrocytes were observed in earlier studies [5,24].

The current study underscores the addition of GFC along with corticosteroids to outperform HSA in significantly improving cell proliferation and cell migration in both ACL-derived stromal cells and chondrocytes.

Thus, acellular therapies like GFC administration along with corticosteroids can alleviate damage to connective tissue cells in musculoskeletal injuries. In agreement, a recent meta-analysis has shown that PRP and PRP combined with hyaluronic acid were efficient in improving function and alleviating pain after three, six, and 12 months of follow-up in knee osteoarthritis patients [28].

Besides cell damage, differences in collagen I staining, a major component of connective tissues, were observed. Similar to our results, dexamethasone-treated tenocytes were shown to decrease the expression of various collagens including collagen I. Furthermore, altered collagen expression was observed in chondrocytes treated with methylprednisolone. Our data from cell phenotypes and recovery of organelle damages corroborate a clinical study where PRP injections were shown to decrease joint pain and improve activity of daily living and quality of life in osteoarthritis as compared to corticosteroids [29]. Furthermore, the data from the current study support our clinical trial data reported earlier using GFC prepared using the Ossinext^TM^ kit. We showed that GFC-treated knee osteoarthritis patients had significantly better improvement in the WOMAC score compared to hyaluronic acid-treated patients, without any increase in the risk of adverse events. GFC showed highly significant (P < 0.0001) improvements in before treatment versus after treatment in the WOMAC, KOOS, IKDC, and VAS scales used for the assessment of knee osteoarthritis.

Limitations

Our study lacked physiological methylprednisolone concentrations and investigated limited samples. Our study also did not determine the critical time periods for administering GFC post-corticosteroid treatment to elicit its cytoprotective effects. Further studies with large cohorts and animal studies for timing GFC administration are required. In addition, randomized controlled trials will be helpful in assessing the effectiveness, safety, and clinical significance.

## Conclusions

GFC prepared from healthy volunteers improves cell proliferation, cell viability, collagen I expression, and organelle damage caused by methylprednisolone. The use of GFC alongside corticosteroids such as methylprednisolone shows promise for improving the healing of ligaments and cartilage. These findings imply potential clinical applications where such combinations could be used to enhance the efficacy of treatments targeting musculoskeletal injuries.
